# Healing of Diabetic Foot Ulcers in Patients Treated at the Copenhagen Wound Healing Center in 1999/2000 and in 2011/2012

**DOI:** 10.1155/2019/6429575

**Published:** 2019-09-08

**Authors:** Marie Louise Buhl Sørensen, Rasmus Bo Jansen, Therese Wilbek Fabricius, Bo Jørgensen, Ole Lander Svendsen

**Affiliations:** ^1^Department of Endocrinology I, Copenhagen Diabetes Foot Center, Bispebjerg Hospital, University of Copenhagen, DK-2400 Copenhagen, Denmark; ^2^Department of Endocrinology I, Bispebjerg Hospital, DK-2400 Copenhagen, Denmark; ^3^Department of Endocrinology, Nordsjællands Hospital, DK-3400 Hillerød, Denmark; ^4^Copenhagen Diabetes Foot Center, Bispebjerg Hospital, DK-2400 Copenhagen, Denmark

## Abstract

**Aim:**

To describe differences in healing time of diabetic foot ulcers for patients treated at the Copenhagen Wound Healing Center, Bispebjerg Hospital, between the years 1999/2000 and 2011/2012. The Center is highly specialized and receives diabetes patients with hard-to-heal foot ulcers. A further aim is to attempt to find predictors of healing time of diabetic foot ulcers.

**Methods:**

A retrospective descriptive study of records from patients with diabetic foot ulcer treated at the Copenhagen Wound Healing Center in 1999, 2000, 2011, or 2012. Follow-up data was collected until the 3^rd^ of August 2018.

**Results:**

Median time (range) to healing was 6 (61.3) months in 1999/2000 and 6.6 (67.8) in 2011/2012 (*p* = 0.2). About 33% of ulcers were healed, 17% were minor or major amputated, and 1.5% were dead within one year in 1999/2000, whereas 30% of ulcers were healed (*p* = 0.6), 14% were amputated (*p* = 0.2), and 12.8% were dead within one year in 2011/2012 (*p* < 0.001). The single factor found significantly associated with longer ulcer duration was infection. Related to shorter ulcer duration were toe localization of the ulcer and good glycemic control.

**Conclusion:**

The median time to healing of a diabetic foot ulcer was long, around 6 months and with a high recurrence rate in 1999/2000 as well as in 2011/2012. Some factors were found to be significantly related to healing time, and intervention addressing these may improve the time to heal, although such interpretations must be taken with precaution from the present study and should be proven in randomized prospective intervention trials.

## 1. Introduction

Diabetic foot ulcers (DFU) are severe complications to diabetes mellitus and are associated with a higher mortality [1], a lower quality of life [2], and ultimately life-threatening amputations [3]. With a life incidence of up to 25% in patients with diabetes, the foot ulcers are frequent and dangerous complications to diabetes [3]. Due to the large burden of diabetic ulcers for the individual as well as society, it is relevant to find predictors of the healing time in the context of prophylaxis and treatment.

Several studies have investigated factors related to the healing time of DFU (diabetic foot ulcer). Evidence suggests a possible association between healing time and factors such as HbA_1c_, ulcer size, infection, peripheral artery disease, etiology, and longer duration of diabetes [4–7]. A recent study presented a linear relationship between HbA_1c_ and the healing rate of diabetic foot ulcers (every 1% increase in baseline HbA_1c_ resulted in a decrease of wound area healed per day by 0.028 cm^2^, *p* = 0.02) [4]. The aim of this current study is to examine such possible predictors of diabetic foot ulcer healing time, for example, diabetic retinopathy and nephropathy, distal blood pressures, medication (antibiotics, antidiabetics, and insulin), blood lipids, HbA_1c_, creatinine, urine albumin, surgical procedures related to the chronic ulcer, and age at ulcer debut. The study includes two cohorts of patients with diabetes treated at the Copenhagen Wound Healing Center (CWHC), Bispebjerg Hospital, Denmark, from the years 1999/2000 and 2011/2012. The Center is highly specialized and receives diabetes patients with hard-to-heal foot ulcers from other health care providers. Another aim is to study whether the healing time of DFU and associated number of amputations has changed between those two periods.

## 2. Materials and Methods

### 2.1. Inclusion and Exclusion

**Figure 1:**
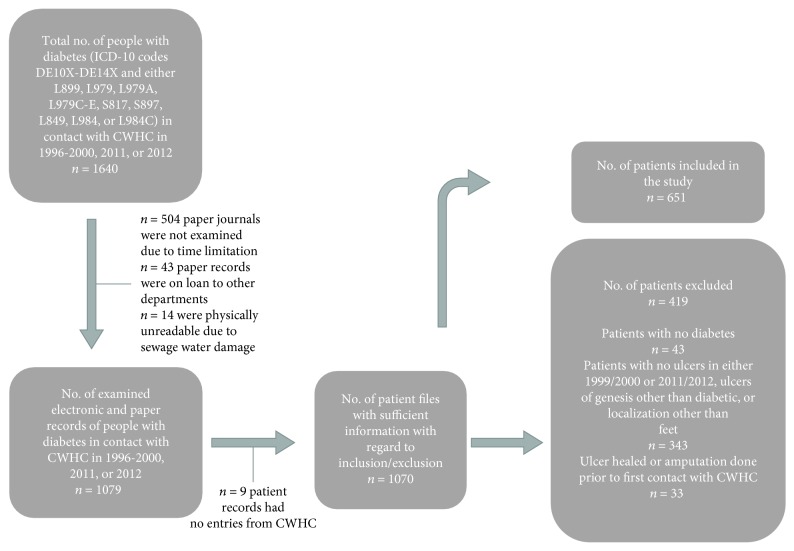
Overview of data collection, inclusion, and exclusion of patients with diabetic foot ulcer.

Medical records of patients with diabetes treated at CWHC at Bispebjerg Hospital in 1999, 2000, 2011, and 2012 were followed up until the 3^rd^ of August 2018. Patients above 18 years of age with ICD-10 codes of diabetes mellitus type 1 or 2 (i.e., DE10X-DE14X) and with diabetic foot ulcer(s) (ICD-10 codes L899, L979, L979A, L979C-E, S817, S897, L849, L984, and L984C) were included in the study, as shown in Figure 1. A list of all the patients with DFU treated at CWHC from 1996 to 2013 were used to find the relevant social security numbers. Patient records from years 1999 and 2000 consisted of records placed in an archive at Bispebjerg Hospital. The paper records are sorted by birthday (from the social security number) of the patients with diabetes. Several had first contact in a year prior to 1999/2000, but still had one or more ulcers in 1999 or 2000, and were therefore included. Inclusion in 2011/2012 was based on the list of all patients with DFUs in contact with CWHC from 1996 to 2013. Records from 2011 and 2012 were found in the electronic patient system, and all of the records of patients in contact with CWHC in these years were assessed (*n* = 556). The information gathered was validated by a coauthor (TW) comparing 6 patient records (3 from 1999/2000 and 3 from 2011/2012) and the information registered for each of them. No significant misinterpretations or mistakes were found. For the sake of convenience, the group of patients treated in 1999 or 2000 will throughout the article be mentioned as only 1999 and the group of patients treated in 2011/2012 as 2012.

### 2.2. Data Recording

The included patients were followed from first contact with CWHC to the last registered record. The patient record prior to the first contact with CWHC was reviewed for any relevant medical procedures (amputations), diagnoses, previous ulcers, and duration of antibiotic treatment going back to 1996, the year that CWHC was founded. Baseline data were registered gender, age, type of diabetes, duration of diabetes, body mass index, smoking and alcohol habits, HbA_1c_, systolic blood pressure, cholesterol, etc. Whether the patient was deceased or not (at the time of inclusion in August 2018) was visible in the electronic patient system but not the death date or reason. Any minor or major amputations were registered.

### 2.3. Description of the Copenhagen Wound Healing Center

The Copenhagen Wound Healing Center is located at Bispebjerg Hospital, is highly specialized, and receives diabetes patients with hard-to-heal foot ulcers from most parts of Denmark. Besides diabetes patients with foot ulcers, the Center treats other patients with chronic wounds, such as venous/arterial leg ulcers, pressure wounds, and more rarely ulcers related to malignancy or immunological disorders. The Center consists of a ward, an outpatient clinic, and a specialized lymphedema-compression clinic. The concept is based on interdisciplinary cooperation between doctors, nurses, foot therapists, physiotherapists, and medical secretaries. Furthermore, the department collaborates with other hospital wards such as Endocrinology, Clinical Physiology, Orthopedic Surgery, and Vascular Surgery (the Copenhagen Diabetes Foot Center).

### 2.4. Data Collection and Definitions

*Diabetes mellitus* was defined as diagnosis with ICD-10 code DE10X-DE14X or if the diagnosis was mentioned in the patient records. *Age* was registered at the debut of the foot ulcer in 1999, 2000, 2011, or 2012, alternatively at the first mention of the ulcer if debut time was unknown. *Debut of the ulcer* was noted as the date when the diabetic foot ulcer was discovered by the patient.

*Alcohol* was registered as weekly intake in units and whether the intake exceeded the Danish National Board of Health's recommendation (<7 units of alcohol per week for women and <14 for men). S*moking* was registered as total number of years of smoking 20 cigarettes a day, daily use of tobacco (grams), and previous smoking habits. *Duration of diabetes* was noted as time of original diabetes diagnosis until debut of the primary foot ulcer—if the primary debut was not found in the patient record, the diabetes duration was noted as the time of original diabetes diagnosis until first mention of the ulcer in question. Both initial and end *HbA_1c_* values were noted, found in the patient records with a margin of about 3 months of the initial debut of the foot ulcer and the time of healing. The *time of healing* and duration of the ulcer were noted. If minor or major amputations were done, this was registered instead of the time of healing—minor amputations being amputations done below ankle level and major amputations above ankle level.

*Peripheral artery disease* (PAD) was defined if the diagnosis was found in the patient record or as either toe blood pressure < 50 mmHg, ankle pressure < 90 mmHg, ankle‐brachial index < 0.90, and/or absence of two foot pulses. If there were ankle‐brachial index values > 1.3 with no/low toe pressure measurements, it was considered unknown whether the person had PAD, due to the possibility of unreliable distal pressure measurements. If the ankle-brachial index pressures were the only distal pressures registered, PAD status was similarly considered unknown.

*Venous insufficiency* was defined as clinical signs (varices, hyperpigmentation, stasis dermatitis, crural edema, and/or venous ulcer) or treatment of venous insufficiency such as surgery. *Ischemic heart disease* was defined when diagnosed with ischemic heart disease, if with clinical signs of angina pectoris, or if with history of acute myocardial infarction or ischemic heart surgery such as CABG. *Nephropathy* was noted if there was a medical record of macroalbuminuria (>300 mg/24 hours or urine albumin/creatinine ratio > 300 mg/g) or an increase in p-creatinine above twice the normal range with the absence of other kidney diseases (two such values should be found at least 1 month apart to ensure chronicity). Additionally, dialysis was considered as an indication of chronic nephropathy. *Retinopathy* was registered if it was diagnosed or if there were signs from fundus photography or ophthalmoscopy of exudates, hemorrhages, micro aneurisms, intraretinal microvascular abnormalities (IrMAs), or vessel proliferation. The definition of *peripheral neuropathy* was sensibility loss by Semmes-Weinstein monofilament examination, >25 volts at biothesiometry, or diagnosis registered in medical files. If a medical history of *cerebral infarction* was found, it was registered. *Infection* was regarded as present if there was a positive finding of bacteria after a wound swab. The accumulated minimum duration of antibiotic treatment by CWHC was counted.

Besides the elements mentioned above, other factors that were registered are age at ulcer debut, location and number of DFU(s), gender, body mass index, type of diabetes, systolic blood pressure, triglycerides, total cholesterol, serum-creatinine, urine albumin, and medication (statins, basal insulin, bolus insulin, antidiabetics, antihypertensive medication, etc.).

### 2.5. Statistical Analysis

Values are given as numbers or median (range) unless otherwise noted. The statistical analysis program used was IBM SPSS Statistics version 22. The association between the main outcomes (ulcer healing, amputation, and death) and categorical variables was assessed via chi-square statistic. Binary logistic regression was used with the same outcomes and continuous or multiple categorical variables. The outcome of time to healing and categorical variables was examined with the use of the Mann-Whitney *U* test. All calculations are based on the first DFU that the patients were treated for at CWHC in either 1999 or 2012, unless otherwise specified in the text. The healing time outcome is also based on a single ulcer per patient. Statistical significance was set at 0.01 (two-sided) to take into account the risk of mass significance.

## 3. Results and Discussion

### 3.1. Excluded Patients

419 of the patients were excluded. Reasons for exclusion were no diabetes diagnosis; no ulcer(s) in the years of 1999, 2000, 2011, or 2012; ulcers with a genesis other than diabetic (such as venous insufficiency, lymphedema, and vasculitis); and ulcers with localization other than the feet (for example shin, thigh, and nates). Furthermore, if the ulcer(s) had healed or if amputation was performed prior to first contact with CWHC, the patient was excluded.

### 3.2. Included Patients

651 patients with diabetic foot ulcers treated at CWHC in the years 1999, 2000, 2011, and/or 2012 were included in the study. 74% were men and 26% women. 80% had type 2 diabetes (T2D), 10% had type 1 diabetes (T1D), and 10% was categorized as IDDM (insulin-dependent diabetes mellitus; unknown whether type 1 or insulin-dependent type 2). 27% were current smokers, 61% were nonsmokers, and 12% had unknown tobacco habits. 19% drank more alcohol than the amounts recommended by the Danish National Board of Health. The average age at ulcer debut was significantly lower (*p* < 0.001) for patients with T1D (50 years in 1999 and about 59 years in 2012) than for those with T2D (67 years in 1999 and 70 years in 2012). The duration of diabetes at DFU debut was higher for patients with T1D (32.7 and 36.3 years, respectively) than for patients with T2D (12.3 and 13.0 years, respectively) in 1999 and in 2012. 48% had ulcer(s) before, 81% had detectable neuropathy, and 59% had arterial insufficiency. The median HbA_1c_ at ulcer debut was 7.8% and at ulcer healing 7.5% (*p* = 0.02). Median BMI was 27.3 (40.9). There was no significant difference in the median number of minor amputations per patient in 1999 and 2012 in T2D (both years with a median of 0 (1) amputations, *p* = 0.09), but a significant difference was found for the median number of minor amputations per patient in 1999 (0 (1)) and 2012 (0(0)) in T1D (*p* = 0.002). More patients with T1D than with T2D had diabetic nephropathy, retinopathy, and neuropathy at baseline. More patients with T2D than with T1D had ischemic heart disease. There were significantly fewer T2D patients with neuropathy in 2012 (70%) than in 1999 (78%, *p* = 0.001).

The median HbA_1c_ at ulcer debut was significantly lower in T2D in 2012 (7.3% (8.7)) than in 1999 (7.9% (10.2), *p* = 0.002). Furthermore, the median HbA_1c_ was significantly lower in T2D than in T1D (10.25% (5.8) in both 1999 (*p* = 0.001) and 2012 (T1D median 9% (8.9), *p* = 0.001)). Also, at healing time of the ulcer, HbA_1c_ was lower in T2D than in T1D in 2012 (7% (11.4) vs. 8.5% (6.9), *p* = 0.006). An intake of statins was more prevalent in 2012 (55%) than in 1999 (7%). Likewise, the intake of antihypertensives was significantly more predominant in 2012 (82%) than in 1999 (50%) in T2D (*p* < 0.001).

### 3.3. Follow-Up

Median follow-up time for the patients with DFU(s) treated at CWHC in 1999 was 5.3 years (19.4) and 3.7 years (8.1) for the ones treated in 2012 (*p* < 0.001). 59% had one or several recurrences of diabetic foot ulcers, and the median was 1 (40) ulcer in the follow-up period. 17% of all the included patients developed chronic nephropathy, 32% did not, and it was unidentified for the remaining (no urine albumin, creatinine, or dialysis was found in the patient record).

A total of 62 patient records had no available data in the electronic patient system and were therefore completely lost to follow-up. When examining for differences in baseline data between the group that was lost to follow-up and the patients that were followed up (*n* = 589), the 62 patients were significantly older at ulcer debut (median age 77 (61) years vs. 67 (72) years, *p* < 0.001) and the percentage of women was higher (40% women vs. 25% women, *p* = 0.008).

### 3.4. Ulcer Characteristics

A total of *n* = 1048 ulcers were found. Every patient developed a median of 1 (7) ulcer which was similar in 1999 and 2000. The median number of days a person had ulcer before contact with CWHC was 57 days (2192). The median surface area within one month of ulcer debut (the size was registered within the first month after debut for 153 of the ulcers, out of all of the ulcers *n* = 1048) was 2 (120) cm^2^. 48.8% were toe ulcers, 21.8% plantar ulcers, 17.8% heel ulcers, and 8.9% dorsal/lateral foot ulcers. Out of the cases with known etiology (*n* = 627), 73% were neuroischemic wounds, 8.5% ischemic, 17.4% neuropathic, and 1.1% allegedly had neither neuropathy nor ischemia.

### 3.5. Outcomes

**Figure 2:**
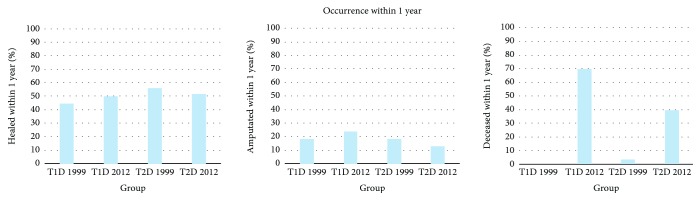
The graphs show occurrence within one year of the outcomes healing, amputation, and death, respectively, in 1999 and 2012 for both T1D and T2D patients.

Figure 2 shows the fraction of healed ulcers, amputations, and deceased within one year. 33% of the ulcers treated in 1999 achieved healing within one year, and 30% treated in 2012 achieved healing within one year (*p* = 0.6). 13% had minor amputations done of the group treated in 1999 within 1 year of ulcer debut and 11% of the patients treated in 2012 (*p* = 0.3). 4% had major amputation done in 1999 and 3% in 2012 (*p* = 0.4).

**Figure 3:**
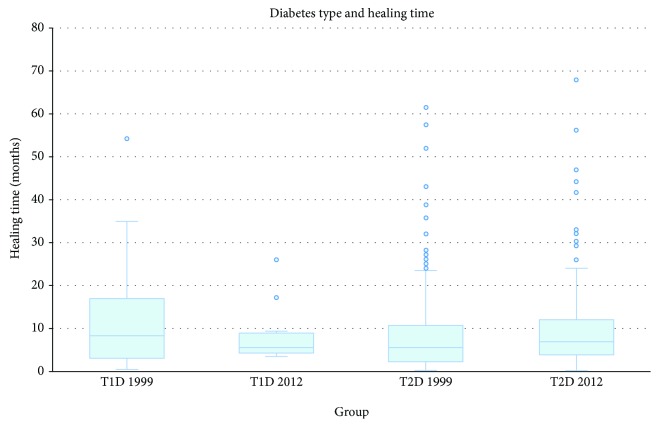
Box plot (with median and quartiles) and scatter plot of the ulcer healing time in months for T1D and T2D patients in 1999 and 2012.

The median (range) healing time was 6 (61.3) months in 1999 and 6.6 (67.8) months in 2012 (*p* = 0.2), as shown in Figure 3. Of the patients treated in 1999, 38.6% experienced one or more recurring diabetic foot ulcers within 12 months. Within a 3-year follow-up, 53% had had a recurrence of ulcer(s). In the 2012 group, 30.3% of the patients had ulcer recurrence within 12 months (*p* = 0.04). Within 3 years, 47% had had recurrence (*p* = 0.09). More men than women experienced relapse of DFU (69% versus 51%, *p* < 0.001). Neuropathy was significantly related to a higher risk of ulcer recurrence (RR = 3.3, *p* = 0.001). Other factors related to recurring DFUs were treatment with antibiotics for more than 3 months (RR = 1.5, *p* < 0.001), >65 years old at primary ulcer debut (RR = 1.6, *p* < 0.001), and having had ulcers before (RR = 1.3, *p* = 0.006).

### 3.6. Factors related to Outcome

Table 1 shows the healing time of DFUs (months) in those with or without risk factors in 1999 and 2012. The calculations are based on the first ulcer each patient had at first contact with CWHC.

Infection was significantly associated with a longer duration of DFU in 1999. In 1999, toe location of the DFU was related to a shorter healing time, and there was a significant longer duration of toe ulcers in 2012 compared to 1999. Infection, smoking 20 cigarettes/day for >20 years, and HbA_1c_ > 7% were all significantly associated with a higher risk of amputation.

None of the procedures that the included patients underwent (Achilles elongation, tenotomy, surgical revision, resection, skin transplants, and/or revascularization) was significantly associated with ulcer duration. There was no significant association between etiology of the ulcer and healing time either. Patients with ulcer for ≤59 days before first contact with CWHC had a median healing time of 4.5 (43) months, and patients with ulcer for ≥60 days had a median healing time of 10.3 (67) months (*p* = 0.1). Patients treated in 1999 had had ulcer for a significantly shorter amount of time (median of 37 days (2192)), before first contact with CWHC, than those treated in 2012 (median of 60 days (1827), *p* = 0.005).

As shown in Table 2, intake of statins, metformin, and minor amputations was related to a lower risk of death, whilst intake of antibiotics for more than 13 months and major amputations showed association with a higher mortality risk.

### 3.7. Discussion

There is a majority of males in this cohort (74% men and 26% women), which has been observed in other studies as well [8–10]. Men seem to develop DFUs more often than women, a tendency that could be related to differences in health behavior: frequency of doctor visits, self-care, compliance, etc.

We found a median duration of ulcer of 6 months in 1999 and 6.6 months in 2012, which is a relatively long time compared to five other DFU studies, which showed mean or median healing times of 1-4 months [5–8, 11].

The healing time had not improved in 2012 compared to 1999. The reason for the comparatively long ulcer duration could be a more progressed disease state of the patients with diabetes treated at CWHC, since it is a specialized unit that receives diabetes patients with several comorbidities and foot ulcers that are difficult to heal. However, the number of amputations were not greater than previously reported in diabetes patients with foot ulcer with less severe disease at the moment of presentation to the foot clinic [12]. A large German study of patients with diabetes found a decrease in the amount of amputations (due to neuropathic, vascular, traumatic, and cancer-related causes) performed on diabetes patients from 2008 to 2012 [13], a tendency also observed in our study between the years of 1999 and 2012, although not statistically significant.

The following factors in our study showed no association to ulcer duration: ischemic heart disease, diabetic nephropathy, retinopathy, venous insufficiency, cerebral infarction, intake of statins, intake of antidiabetics (such as DPP-IV blockers, glinides, sulfonylureas, GLP-1 agonists, or metformin), insulin treatment, antihypertensives, urine albumin, creatinine, blood lipids, systolic blood pressure, alcohol (more than the recommended amount), being a former smoker, type of DM, duration of DM, BMI, no. of ulcers at ulcer debut, age at ulcer debut, no. of former amputations, no. of former ulcers, ulcer area, smoking, intake of antibiotics, gender, and procedures (Achilles elongation, tenotomy, surgical revision, resection, skin transplants, and/or revascularization).

The factors that we found to be significantly associated with a longer ulcer duration were infection and toe localization of the ulcer. Infected ulcers have previously been connected to prolonged ulcer healing time [14, 15] and toe localization to shorter healing time compared to the mid and the hind foot [5, 16].

Similar to what other studies have observed, we found a significant association between a lower chance of healing and HbA_1c_ > 7% [17]. Like other studies, our results show a bigger risk of amputation related to infection, smoking 20 cigarettes/day in >20 years, and HbA_1c_ > 7% [18–21].

In our study, the patients had had ulcer for 57 days on average before first contact with CWHC, which seems to confirm that our patient population has a more advanced disease with foot ulcers that are hard to heal at first contact with CWHC. In the Eurodiale study [22], more than 1/4 of the patients with a diabetic foot ulcer were treated elsewhere for more than 3 months, before referral to a specialized foot clinic. As emphasized by international guidelines, quicker referral to specialized foot clinics is needed.

Previously, it was common practice at CWHC to treat diabetes patients with long-lasting open ulcers with antibiotics as long as osteomyelitis and probe-to-bone contact were present, which for some of the patients in our study lasted for more than 13 months. Although there is no or little evidence on how long antibiotics should be given in these cases [23], long-term treatment with antibiotics is not recommended. Given the data in this study, in the future, we will carefully (re)consider the indication for continued use of antibiotics. Randomized clinical trials are needed.

Treatment with antibiotics > 13 months and undergoing major amputations were associated with an increased risk of death. This is consistent with results from other studies that show a relation between infected ulcers and a higher risk of major amputation and major amputation being associated with a higher mortality risk [23, 24]. Oppositely, metformin and statins were related to a lower risk of death in accordance with results from other studies that link metformin to a lower all-cause mortality [25] and statins with a reduced risk of cardiovascular mortality in people with diabetes [26, 27]. In this study, an intake of statins was more prevalent in 2012 (55%) than in 1999 (7%) most likely due to the introduction of routinely used statins in diabetic treatment (*p* < 0.001).

Several other studies have focused on the healing process of diabetic foot ulcers, fewer of these on the healing time. One of the strengths to this study is patient data going 22 years back in time and including more than 600 patients. Only one other study had a study period of roughly the same amounts of years (21 years); however, that study focused on plantar forefoot ulcers only and did not include dorsal foot ulcer, toe ulcers, or heel ulcers [8]. One of the limitations of the study was the relatively large amount of information that was not obtainable from the patient records. Another limitation was the relatively short follow-up time period despite the study going back 22 years in time (1996 until 2018), which could be explained by the fact that the majority of included patients were at some point in time lost to follow-up for reasons unknown (no more hospital contacts, emigration, death, etc.). Prospective capture of clinical data on wound healing by use of prospective clinical databases is needed.

## 4. Conclusions

Our study showed that the median time to healing of a diabetic foot ulcer was long, around 6 months, and with a high recurrence rate in 1999/2000 as well as in 2011/2012. These outcomes should be considered taking into account that the patients with diabetes referred to CWHC are patients with a more progressed disease state, comorbidities, and foot ulcers that are hard to heal.

The single factor found negatively related to ulcer healing was infection. Factors found to be positively connected to ulcer healing were good glycemic control and toe localization. Our results support the general view that treating infections and seeking treatment early on when noticing a newly forming ulcer are important steps in treating diabetic foot ulcers. Intervention addressing these may improve the healing time of diabetic foot ulcers, although further evidence from randomized prospective intervention trials is needed.

## Figures and Tables

**Table 1:** Risk factors affecting healing time in chronic diabetic foot ulcers.

Risk factors and average ulcer duration	1999/2000	*n* ^5^ = 160 (324)	*p* value^1^	*p* value^2^	2011/2012	*n* ^5^ = 148 (327)	*p* value^3^	*p* value^4^
Median (range), months	**+**	-			**+**	-		
Peripheral arterial insufficiency	6.0 (60) *n*^6^ = 80	5.3 (53.8) *n* = 40	0.2	0.5	6.0 (60) *n* = 80	7.8 (30.5) *n* = 16	0.4	0.1

Ischemic heart disease	8 (35) *n* = 25	5.9 (61.3) *n* = 113	0.4	0.6	6.9 (55) *n* = 38	6.6 (67.8) *n* = 106	0.7	0.2

Neuropathy	5.9 (61.3) *n* = 134	6.4 (24.5) *n* = 6	0.9	0.3	6.5 (67.8) *n* = 115	5.3 (10.5) *n* = 10	0.3	0.8

Chronic nephropathy	10.4 (50.5) *n* = 20	7.5 (61.3) *n* = 53	0.4	0.3	4.3 (40.3) *n* = 21	7.0 (67) *n* = 95	0.6	1.0

Retinopathy	9.0 (51.8) *n* = 30	5.5 (24.3) *n* = 21	0.2	0.9	7.3 (54.8) *n* = 35	5.6 (25.0) *n* = 30	0.2	0.8

Metformin	6.5 (57) *n* = 22	7.3 (61.3) *n* = 108	0.3	0.7	6.0 (67.8) *n* = 81	6.8 (43.8) *n* = 65	0.6	0.9

Basal insulin	6.8 (61.3) *n* = 60	7.0 (57.0) *n* = 78	0.9	0.4	7.1 (66.5) *n* = 60	6.3 (56) *n* = 87	0.5	0.8

Bolus insulin	7.1 (54) *n* = 40	7.0 (61.0) *n* = 92	0.9	0.8	6.6 (40.3) *n* = 38	6.5 (67.8) *n* = 107	0.9	0.9

Infection	9.0 (61.0) *n* = 77	3.5 (17.5) *n* = 29	<0.001^∗^	0.4	7.4 (67.8) *n* = 86	5.1 (4.3) *n* = 4	0.4	0.3

Distal pulse, right ADP	6.0 (61.0) *n* = 65	7.8 (60) *n* = 52	0.07	0.03	7.0 (67.0) *n* = 75	7.4 (56) *n* = 56	0.8	0.9

Former ulcer(s)	6.0 (61.3) *n* = 87	7.0 (57.0) *n* = 55	0.9	0.08	8.1 (55.8) *n* = 56	6.3 (67.8) *n* = 65	0.1	0.9

Smoking	7.3 (60.8) *n* = 45	5.4 (60) *n* = 92	0.03	0.4	6.8 (42.8) *n* = 34	6.5 (56) *n* = 107	0.8	0.07

Type 2 DM^∗∗^	5.8 (61.3) *n* = 119	7.9 (53.8) *n* = 20	0.3	0.08	6.7 (67.8) *n* = 134	5.5 (22.5) *n* = 11	0.8	0.9

Male gender	6.8 (61.3) *n* = 124	5.1 (26.8) *n* = 36	0.3	0.3	7.0 (67.5) *n* = 115	5.3 (56.0) *n* = 33	0.4	0.5

Antibiotics	6.6 (61.3) *n* = 134	5.6 (16.5) *n* = 16	0.5	0.4	7.3 (67.8) *n* = 99	4.8 (40.3) *n* = 33	0.3	0.9

Toe location of ulcer	4.9 (42.8) *n* = 64	9 (61) *n* = 68	0.001^∗^	0.007^∗^	7.0 (67.0) *n* = 59	8.5 (55.3) *n* = 73	0.5	0.7

Heel location	8.0 (59.5) *n* = 20	6.8 (61.3) *n* = 112	0.2	0.5	8.3 (55.3) *n* = 28	7.9 (67.0) *n* = 104	1.0	0.06

Plantar location	8.7 (61) *n* = 42	5.4 (60) *n* = 90	0.03	0.6	10 (46.0) *n* = 33	7.3 (67.0) *n* = 99	0.07	0.07

Dorsal location	9.7 (33.3) *n* = 6	6.8 (61.3) *n* = 126	0.4	0.3	5.8 (16.0) *n* = 12	8.0 (67.0) *n* = 120	0.1	0.05

The table shows the median and the range. The calculation is based on the first ulcer each patient had at the time of first contact with CWHC (*n* = 651). ^∗^*p* < 0.01. ^∗∗^Compared to type 1 DM. ^1^*p* value comparing “+” and “-” in 1999/2000. ^2^Comparing “+” in years 1999/2000 and 2011/2012. ^3^Comparing “+” and “-” in 2011/2012. ^4^Comparing “-” in years 1999/2000 and 2011/2012. ^5^Total number of ulcers with known duration. Parenthesis: the total number of patients with DFUs in 1999/2000 or 2011/2012. ^6^*n* is the number of ulcers with known duration.

**Table 2:** Risk factors and mortality. Based on all the included patients (*n* = 651).

Deceased no. (% of patients with nonmissing data)	**+**	-	*p* value	OR	RR
Statins	120 (20.5) *n* = 585	303 (51.8) *n* = 585	<0.001	0.3	0.6

Metformin	101 (17.3) *n* = 585	323 (55.2) *n* = 585	<0.001	0.3	0.6

Minor amputation(s) done	77 (16.2) *n* = 647	399 (83.8) *n* = 647	0.01	0.6	0.9

Major amputation(s) done	40 (6.2) *n* = 647	436 (67.4) *n* = 647	0.007	15.4	1.1

>13 months of antibiotic treatment	58 (10.0) *n* = 582	375 (64.4) *n* = 582	0.002	4.5	1.1

*n* is the total no. of patients with nonmissing data.

## Data Availability

The data is available at the site (Copenhagen Wound Healing Center) by inquiry to the first author via email (marie.louise.buhl.soerensen@regionh.dk).

## References

[1] Walsh J. W., Hoffstad O. J., Sullivan M. O., Margolis D. J. (2016). Association of diabetic foot ulcer and death in a population-based cohort from the United Kingdom. *Diabetic Medicine*.

[2] Hogg F. R. A., Peach G., Price P., Thompson M. M., Hinchliffe R. J. (2012). Measures of health-related quality of life in diabetes-related foot disease: a systematic review. *Diabetologia*.

[3] Singh N., Armstrong D. G., Lipsky B. A. (2005). Preventing foot ulcers in patients with diabetes. *JAMA*.

[4] Christman A. L., Selvin E., Margolis D. J., Lazarus G. S., Garza L. A. (2011). Hemoglobin A1c predicts healing rate in diabetic wounds. *The Journal of Investigative Dermatology*.

[5] Ince P., Kendrick D., Game F., Jeffcoate W. (2007). The association between baseline characteristics and the outcome of foot lesions in a UK population with diabetes. *Diabetic Medicine*.

[6] Zimny S., Schatz H., Pfohl M. (2002). Determinants and estimation of healing times in diabetic foot ulcers. *Journal of Diabetes and its Complications*.

[7] Messenger G., Masoetsa R., Hussain I., Devarajan S., Jahromi M. (2018). Diabetic foot ulcer outcomes from a podiatry led tertiary service in Kuwait. *Diabetic Foot & Ankle*.

[8] Orneholm H., Apelqvist J., Larsson J., Eneroth M. (2015). High probability of healing without amputation of plantar forefoot ulcers in patients with diabetes. *Wound Repair and Regeneration*.

[9] Zhang P., Lu J., Jing Y., Tang S., Zhu D., Bi Y. (2017). Global epidemiology of diabetic foot ulceration: a systematic review and meta-analysis. *Annals of Medicine*.

[10] Hangaard S., Rasmussen A., Almdal T. (2019). Standard complication screening information can be used for risk assessment for first time foot ulcer among patients with type 1 and type 2 diabetes. *Diabetes Research and Clinical Practice*.

[11] Jeffcoate W. J., Chipchase S. Y., Ince P., Game F. L. (2006). Assessing the outcome of the management of diabetic foot ulcers using ulcer-related and person-related measures. *Diabetes Care*.

[12] van Battum P., Schaper N., Prompers L. (2011). Differences in minor amputation rate in diabetic foot disease throughout Europe are in part explained by differences in disease severity at presentation. *Diabetic Medicine*.

[13] Claessen H., Narres M., Haastert B. (2018). Lower-extremity amputations in people with and without diabetes in Germany, 2008–2012 – an analysis of more than 30 million inhabitants. *Clinical Epidemiology*.

[14] Ndosi M., Wright-Hughes A., Brown S. (2018). Prognosis of the infected diabetic foot ulcer: a 12-month prospective observational study. *Diabetic Medicine*.

[15] Kee K. K., Nair H. K. R., Yuen N. P. (2019). Risk factor analysis on the healing time and infection rate of diabetic foot ulcers in a referral wound care clinic. *Journal of Wound Care*.

[16] Pickwell K. M., Siersma V. D., Kars M., Holstein P. E., Schaper N. C., on behalf of the Eurodiale consortium (2013). Diabetic foot disease: impact of ulcer location on ulcer healing. *Diabetes/Metabolism Research and Reviews*.

[17] Musa H. G., Ahmed M. E. (2012). Associated risk factors and management of chronic diabetic foot ulcers exceeding 6 months’ duration. *Diabetic Foot & Ankle*.

[18] Beaney A. J., Nunney I., Gooday C., Dhatariya K. (2016). Factors determining the risk of diabetes foot amputations--a retrospective analysis of a tertiary diabetes foot care service. *Diabetes Research and Clinical Practice*.

[19] Mantovani A. M., Fregonesi C. E. P. T., Palma M. R., Ribeiro F. E., Fernandes R. A., Christofaro D. G. D. (2017). Relationship between amputation and risk factors in individuals with diabetes mellitus: a study with Brazilian patients. *Diabetes & Metabolic Syndrome: Clinical Research & Reviews*.

[20] Zhou Z. Y., Liu Y. K., Chen H. L., Yang H. L., Liu F. (2015). HbA1c and lower extremity amputation risk in patients with diabetes: a meta-analysis. *The International Journal of Lower Extremity Wounds*.

[21] Wang N., Yang B. H., Wang G. (2019). A meta-analysis of the relationship between foot local characteristics and major lower extremity amputation in diabetic foot patients. *Journal of Cellular Biochemistry*.

[22] Siersma V., Thorsen H., Holstein P. E. (2013). Importance of factors determining the low health-related quality of life in people presenting with a diabetic foot ulcer: the Eurodiale study. *Diabetic Medicine*.

[23] Lipsky B. A., Aragón-Sánchez J., Diggle M. (2016). IWGDF guidance on the diagnosis and management of foot infections in persons with diabetes. *Diabetes/Metabolism Research and Reviews*.

[24] Thorud J. C., Plemmons B., Buckley C. J., Shibuya N., Jupiter D. C. (2016). Mortality after nontraumatic major amputation among patients with diabetes and peripheral vascular disease: a systematic review. *The Journal of Foot & Ankle Surgery*.

[25] Campbell J. M., Bellman S. M., Stephenson M. D., Lisy K. (2017). Metformin reduces all-cause mortality and diseases of ageing independent of its effect on diabetes control: a systematic review and meta-analysis. *Ageing Research Reviews*.

[26] Pyörälä K., Pedersen T. R., Kjekshus J. (1997). Cholesterol lowering with simvastatin improves prognosis of diabetic patients with coronary heart disease: a subgroup analysis of the Scandinavian Simvastatin Survival Study (4S). *Diabetes Care*.

[27] Heart Protection Study Collaborative Group (2003). MRC/BHF heart protection study of cholesterol-lowering with simvastatin in 5963 people with diabetes: a randomised placebo-controlled trial. *The Lancet*.

